# Qualitative Study on Safe and Effective Handover Information during a Rapid Response Team Encounter

**DOI:** 10.1097/pq9.0000000000000650

**Published:** 2023-05-22

**Authors:** Justin M. Greenberg, Anita Schmidt, Todd P. Chang, Alyssa Rake

**Affiliations:** From the * Department of Anesthesia and Critical Care Medicine, Children’s Hospital Los Angeles, Los Angeles, Calif.; † Department of Emergency Medicine, Children’s Hospital Los Angeles, Los Angeles, Calif.

## Abstract

**Introduction:**

A rapid response team (RRT) evaluates and manages patients at risk of clinical deterioration. There is limited literature on the structure of the rapid response encounter from the floor to the intensive care unit team. We aimed to define this encounter and examine provider experiences to elucidate what information healthcare staff need to safely manage patients during an RRT evaluation.

**Methods:**

This phenomenological qualitative study included 6 focus groups (3 in-person and 3 virtually) organized by provider type (nurses, residents, fellows, attendings), which took place until thematic saturation was reached. Two authors inductively coded transcripts and used a quota sampling strategy to ensure that the focus groups represented key stakeholders. Transcripts were then analyzed to identify themes that providers believe influence the RRT’s quality, efficacy, and efficiency and their ability to manage and treat the acutely decompensating pediatric patient on the floor.

**Results:**

Transcript coding yielded 38 factors organized into 8 themes. These themes are a summary statement or recap, closed-loop communication, interpersonal communication, preparation, duration, emotional validation, contingency planning, and role definition.

**Conclusions:**

The principal themes of utmost importance at our institution during an RRT encounter are preparation, a brief and concise handoff from the floor team, and a summary statement from the intensive care unit team with contingency planning at the end of the encounter. Our data suggest that some standardization may be beneficial during the handoff.

## INTRODUCTION

In the inpatient setting, the rapid response team (RRT) is an interdisciplinary group of providers that evaluates and manages patients at risk of clinical deterioration, ultimately deciding if the patient requires transfer to the Intensive Care Unit (ICU).^1–6^ A RRT decreases morbidity, mortality, length of stay, and in-hospital cardiopulmonary arrest.^1–4,6–9^ Data regarding the effectiveness of pediatric-specific RRTs and their effect on mortality are conflicting. Two of the most cited pediatric studies on RRTs, 1 by Sharek et al^6^ and 1 by Brilli et al,^1^ highlight the necessity of a RRT to minimize cardiac or respiratory compromise outside the ICU. Kutty et al, however, found no improvement in mortality with the implementation of an RRT (termed medical emergency team, or MET); they suggested that 1 limitation to MET effectiveness is the suboptimal implementation of the MET, proposing that better protocols and implementation could improve mortality.^10^ The MET (or RRT) process needs further investigation.

RRT evaluations are fraught with unique challenges. One is the limited time available for the interaction between the ICU and the primary team.^11,12^ This fact contrasts with the typical medical consultation, where providers have time to evaluate all aspects of patient care before incorporating literature-based protocols into their treatment recommendations.^13^ The RRT also includes the challenge of triaging the acutely decompensating patient, which involves quickly initiating therapies while obtaining a detailed yet concise handoff from the care team concurrently. Although some RRT encounters are consults for treatment, most RRT encounters arise when patients are actively deteriorating and need immediate intervention and transfer.^8^ The heterogeneity of the patient diagnoses that require RRT evaluation underscores the need for a good handoff.

There is limited literature on the structure of RRT handoffs from the floor to the ICU team in pediatric patients.^14,15^ Standardized handoffs [by using a tool such as situation, background, assessment, and recommendation (SBAR)] have been studied extensively in other medical environments, including nursing,^16–20^ Emergency Medical Services personnel to Emergency Department providers,^9,21,22^ and from the operating room team to the Postanesthesia Care Unit (PACU) team.^23,24^ Given the unique characteristics of the RRT, as discussed, these standardized handoffs may not capture the most important information needed.

Our institution’s critical care medicine department has suggested that variability in communication during an RRT activation makes it difficult to quickly synthesize an appropriate assessment and plan when caring for an acutely decompensating patient. This issue has led to general dissatisfaction with the handover process and has sometimes limited the ICU team’s ability to efficiently care for the acutely decompensating patient. To create efficient pediatric RRTs, the elements that make them successful must be understood. Our phenomenological qualitative study aimed to uncover which factors must be communicated between the floor and ICU teams to ensure the successful transition of care during a RRT evaluation.

## METHODS

We conducted a phenomenological qualitative study from February to October 2020 at a single-center, tertiary-care, free-standing children’s hospital in an urban environment. This design was chosen because there are limited data regarding what comprises a successful handoff in this setting, and because we aimed to capture the perspectives of the interdisciplinary group involved in the RRT. For reference, approximately 700 RRT activations occurred in 2019 at this hospital. Rapid response activations differ from code blue activations; however, the same team responds to both. Approximately 50% of RRT activations in our institution result in patient transfer to the ICU; the other patients are treated and remain on the ward.

The RRT includes pediatric residents/hospitalists, critical care medicine fellows, pediatric ICU nurses, and respiratory therapists. A provider, a nurse, a respiratory therapist, or a family member can initiate RRT activations. This study utilized the Equator Network Standards for Reporting Qualitative research for its analysis (Standards for Reporting Qualitative research Checklist. http://links.lww.com/PQ9/A480).^25^

We conducted focus groups to obtain comprehensive information directly from key stakeholders and organized them by provider type to encourage participants to speak freely with their colleagues and maximize discussion.^26–28^ This format allowed for group exploration of topics and themes. The Institutional Review Board approved this study.

We used a quota sampling strategy to ensure that the focus groups represented key stakeholders.^29^ These groups included hospital medicine attendings, charge nurses, resident physicians, and critical care medicine fellows. Inclusion criteria comprised being one of the above-listed groups and having participated in 1 or more RRT activations. The focus groups did not include critical care medicine attendings as they typically do not attend the RRT activations unless requested by the critical care medicine fellow. In addition, the authors recruited participants via email, participation was voluntary, and there was no incentive to participate. There were no other eligibility criteria regarding demographic or clinical experience.

The authors developed a semistructured interview guide with open-ended questions to elicit focus group participant perceptions of our institution’s RRT encounter (Table 1). The authors JG (critical care medicine fellow) and AJR (critical care medicine attending) developed the guide based on verbal feedback from the RRT committee and the authors’ clinical experience. The questions were then iteratively modified under author ARS, a nonclinical qualitative research expert. Survey questions included: recall of a well-run RRT encounter, documentation of information important to the stakeholders during an RRT encounter, and group preference for a concise or more thorough handoff.

**Table 1.** Focus Groups Questions

Recall or reflect on one of the last RRTs you attended that went well• Why do you think that particular RRT went well?• Communication (or lack thereof)?• Interaction between teams (Floor and ICU)
Recall or reflect on one of the last RRTs you attended that did not go well• Why do you think that particular RRT did not go well?• How could that RRT have been improved?
What does it mean when an RRT was “run well?”
If you had to favor one side or the other, which would you favor in an ideal RRT, and why?• Favor brevity/ shortness – which could risk insufficient information• Favor comprehensiveness – which could risk extraneous information
What information do you look for during the handoff? (if meeting with fellows)
What information do you aim to provide to the ICU team during the handoff (if meeting with residents or attendings)
Should every RRT be run in the same or similarly? Why or why not?
In your opinion, is there consensus about who is in charge during an RRT?• Should this change if resident or attending is calling the RRT?
How would you or your team use a checklist during an RRT?
How would a tool/ format/ practice/ ritual/ cognitive device improve the RRT experience?• How could it be deployed to make an RRT that did not go well into an RRT that did run well?
Are there nonclinical factors that you consider in how to develop a plan during an RRT?• Time of day/night RRT is called• Bed availability –including other pending admissions from ED, OR, or outside hospital• Trust/comfort level of nursing staff or physician caring for patient• Parental concern/request

Keeping with qualitative research standards, each focus group comprised 4 to 6 members.^30^ To mitigate bias, 1 author, ARS, a nonclinician with extensive experience conducting focus groups, moderated the focus groups.^25,31^ Participants did not know the moderator, allowing them to speak freely without concern that comments would not remain anonymous. Group sessions lasted no more than 1 hour and were audio recorded via cell phone (for in-person focus groups) or through the WebEx application (for the virtual focus groups). We curtailed focus groups after they reached thematic saturation.^30^ The first 3 focus groups were held in-person between February and March 2020. Due to the COVID-19 pandemic and mandated restrictions to in-person gatherings, the last 3 focus groups took place virtually in September and October 2020.^32^

Audio recordings of the focus groups were transcribed by an independent external source (TranscribeMe, Inc., Oakland, Calif.) and coded individually using external software (Microsoft Word) by JG and AJR, who were not present during the focus groups. ARS trained JG and AJR in coding. Both investigators discussed impressions, reviewed notes, and read all transcripts to create a coding template of major themes from the focus groups. The coders piloted coding to identify major variations, assure an understanding of code definitions, and finalize the codebook. The authors analyzed the transcripts as received from the external transcription source rather than all at once. JG and AJR then independently coded the participant responses into thematic categories. Numerous virtual meetings were held between JG and AJR to review the transcription coding, determine consensus, and organize the data into themes.

### Results

To reach thematic saturation, we conducted 6 focus groups (2 of each provider type), 3 in-person and 3 virtually. A total of 35 providers participated. Table 2 lists participant demographics. The codebook contained 38 unique codes organized into 8 overarching themes: preparation, role definition, interpersonal communication, emotional validation, contingency planning, duration, closed-loop communication, and a summary statement. Table 3 summarizes the themes. We discuss the themes in detail below, with quotes taken directly from the focus groups.

**Table 2.** Focus Group Participants Demographics

Provider Type (Total)	PGY year, if applicable	Men	Women
Resident (11)	PGY 1	1	10
Fellow (10)	PGY 4 (2); PGY5 (4); PGY6 (4)	4	6
Ward Attending (8)	N/A	4	4
Ward RN (4)	N/A	1	3

**Table 3.** Summary of 8 Themes

Theme	Description
Summary statement/recap	Recap by the team leader that includes an assessment and plan to confirm that stakeholders have the same shared mental model regarding the patient
Closed-loop communication	Communication among members involved in the ICU consult, possibly using SBAR format (situation, background, assessment, recommendation)
Interpersonal communication/interprofessional trust	Communication among team members to ensure that all team member’s voices are heard and to establish a calm, professional atmosphere, including clear role definition
Preparation	Preparation from the floor team prior to the RRT--recent note printed or designating someone to call consults and to open labs and imaging prior to the ICU team arrival
Acknowledgment	Acknowledgement by the ICU team of the work/preparation done by the floor team prior to RRT, a term we have defined as emotional validation
Contingency planning	Creation of if/then statements for next steps by ICU team, especially if patient to remain on the floor
Duration	Most focus group participants favor brevity, or a concise, brief amount of information provided
Role definition	Clearly assigning a leader from the beginning; who should be the leader? The ICU team member or the primary team?

### Preparation

A recurring theme throughout the 6 focus groups centered around the preparation required before the arrival of the RRT. First, participants felt it helpful if, before initiating a RRT encounter, the floor team printed out the most recent note, had the most recent labs/imaging on a mobile workstation ready for review, and had a team member designated to call consultants. If not done, focus group participants felt the RRT evaluation was disorganized. As 1 resident said, “[have] a printed note…know the detailed history…empower the bedside nurse to present his or her concerns…have a discussion beforehand about what they hope to achieve by calling a rapid response.” A fellow added that “…the description that the resident gave the RRT team about what was happening with the patient was very clear,” making it easier to triage the patient’s acuity and provide the appropriate care.

Further, residents and attending participants felt it helpful for the ICU team to review the patient’s chart before their arrival at the bedside to familiarize themselves with the care the floor team has already provided the patient.

### Role Definition

In general, there was little disagreement amongst the participants of the focus groups. However, 1 exception occurred when discussing role definition and who should be the team leader while managing the patient. Some residents felt that the senior resident should remain the team leader, especially if they had been beside for a significant time before the ICU team arrived. Others felt that the intern should be in charge so that they could learn how to rapidly synthesize and present the information to a larger group of people. Several residents noted that “…whoever called the [rapid response] should be the one [to lead]…” The ICU fellows felt differently, noting, “…they really want you to take over care…they want you to lead the situation” while continuing to take input and advice from all stakeholders.

### Interpersonal Communication/Interprofessional Trust

Participants felt that interpersonal communication among team members was important during the evaluation of a patient by the RRT. It is not uncommon that the ICU team does not know the other staff members present at the RRT encounter and vice versa. Many fellows and residents noted that team introductions at the beginning of the RRT evaluation might help alleviate some of this unfamiliarity between team members, allowing for a more seamless transition of care and increased trust amongst team members when interventions are requested.

Several participants spoke about how difficult and tense RRTs can be when communication and trust are not optimal. One fellow noted sometimes they are “…very emotionally charged. There was a lot of discomfort…[and] there was already an adversarial stance between the RRT team and the floor team before we arrived.” Another fellow added that “… they’re chaotic, a lot of people showing up to the room, going in the room or outside the room. There’s no clear concern that’s identified early on.”

Other participants mentioned strategies that increased trust and communication levels during an RRT. For example, 1 resident physician noted, “[it] is nice when the floor team feels that the PICU team is invested in hearing about the problem that’s going on with sincerity.” Another resident noted that the “fellow that came down was very patient and, kind of, walked through these very complicated patient’s problems with us.”

### Emotional Validation

Participants, particularly the residents and hospitalists, repeatedly mentioned that they appreciate when the ICU team acknowledges the amount of work, preparation, and attempted interventions that the floor team attempted before requesting the assistance of the RRT. However, this does not always occur. As 1 resident noted, “… [the ICU] team was dismissive… I just felt that we weren’t heard enough.” Another resident stated, “…resident concerns and resident distress are not highlighted.”

When asked what made the RRT encounter successful, 1 provider answered that the “PICU really acknowledges the work that the [general pediatrics] team has already done and the things that they’ve attempted to do to improve the situation.” This response was considered especially true for interventions that are not typically floor protocol but were done anyway in anticipation of an ICU evaluation. For example, the floor team may have already given multiple extra doses of albuterol to a patient with an asthma exacerbation, or they may have increased supplemental oxygen therapy for the acutely hypoxemic patient. Another area where emotional validation was mentioned repeatedly was if, after evaluation, the patient was to remain on the floor, but the floor team still had some concerns. One resident noted that they appreciated when the fellow would “[honor] that safe space and allowing people to share their ongoing concerns.”

### Contingency Planning

Many focus group participants felt that contingency planning, including an if/then statement for the next steps, was essential to the process of the RRT evaluation. This addition was most important when the team decided that the patient did not require transfer to the ICU. As 1 resident physician said, “when this child stays, being aware of what the next steps will be for everyone and setting the standard not only for the medical team but also for the nurses who are taking care of these patients on the units.” Another resident commented, “…do some contingency planning so that there is a plan and whether that means the ICU is going to check in or we’re going to monitor for the next 3 hours and hear the specific things that we’re going to look at and do, that if-then kind of scenario planning.”

### Duration

The next major theme elucidated from the focus groups is the duration of the RRT. Most participants favored brevity over verbosity, with concise information provided. As 1 attending physician stated, **“**I’d lean towards brevity and making sure that everyone is staying concise. Certainly, you don’t want to miss details that are important...but no, we don’t need to rehash the entire patient’s life existence up to that point.” However, a few residents and attending participants felt that a longer, more thorough handoff was preferred to ensure no information was left out. Interestingly, none of the ICU fellows who participated in the focus groups felt that a longer handoff was warranted.

### Closed-loop Communication

Participants mentioned that closed-loop communication among the team members involved in the RRT encounter is vital for the initial evaluation and assessment of the patient. This idea emerged in 2 different ways. First, some participants (typically the attending physicians) expressed interest in formalizing this type of communication by using another proven handoff tool, such as I-PASS (Illness severity, patient summary, action list, situation awareness and contingency planning, synthesis by receiver) or SBAR, when discussing the patient with the ICU team to explain the rationale behind the RRT activation. However, as noted by a participant, “being really direct about why we’re activating the RRT and what we’re concerned about, so I think an SBAR approach is actually more appropriate.”

Second, closed-loop communication was vital during the acute management of the rapidly deteriorating patient to ensure that the appropriate treatments and medications were provided in the correct order and doses by offering a concrete plan with the ICU team. One participant recommended asking, “do you understand where we’re going with this plan, and do you have any concerns?”

### Summary Statement

Many participants mentioned that the ICU team leader’s summary statement or recap should be included at the end of the RRT evaluation, especially if the patient did not require transfer to the PICU. Although similar to the concept of contingency planning, they are distinct themes. The summary statement is an all-inclusive recap of the patient and their overall trajectory. In contrast, the contingency planning focused on an if/then statement for the next steps for the patient that remained on the floor. A resident physician suggested that the PICU fellow provide a formal recap after the RRT evaluation “by summarizing what has happened, what the plan is, and has a plan to check in.” Focus group participants suggested that this statement should include a thorough assessment of the patient and care plan, or a follow-up plan, to ensure that all stakeholders have the same shared mental model regarding the patient’s severity of illness and treatment and management decisions; this would ensure that the patient receives the treatment necessary, especially if they stay on the floor.

## DISCUSSION

Our study aimed to understand and define the essential elements of a RRT encounter. We conducted a phenomenological qualitative study using focus groups to comprehensively identify themes that can affect the quality of this encounter and ultimately impact patient care. Eight themes were identified: preparation, role definition, interpersonal communication, emotional validation, contingency planning, duration, closed-loop communication, and a summary statement.

The data presented in the papers by Sanjuan-Quiles et al, Lockwood et al, and Matern et al^33–35^ highlight the need for a standardized handoff process. Malenka et al showed that a standardized handoff protocol from the operating room to the PICU improved transfer of care without increasing the duration, an important theme discussed in the focus groups.^36^ This study provides some key factors to incorporate into a framework for a standardized handoff process, specifically for a rapid response encounter. Interprofessional trust is known to be a major determinant in the success of the RRT. The responses from the focus groups highlight the importance of human interactions and emotions and suggest that they can affect the RRT evaluation. The focus groups identified several factors that directly contribute to interprofessional trust, many of which are actionable, such as the quality of communication, maintaining a calm and collegial environment, and who is included in the conversation. These elements should be standardized across RRT evaluation to optimize the encounter.

In other hospital settings, most commonly the operating room and the PACU, standardized verbal handoffs such as SBAR reduce medical errors and increase the handoff quality.^9,16,20,21,23,36^ Our focus group participants agreed with that assessment, suggesting that some form of verbal and written standardization may be beneficial during the handoff. However, existing handoff tools like SBAR are unlikely sufficient in this scenario. Although there is some overlap of standard information required for any transition of care scenario in the hospital (basic medical history, allergies, the reason for admission/ED visit/surgical history, for example), there is some information required to evaluate and treat the rapidly decompensating patient that is unique. Specifically, our data suggest that preparation followed by a brief and concise handoff from the floor team and a summary statement with contingency planning from the ICU team before the termination of the rapid response evaluation are necessary components of the RRT encounter. In addition, the focus groups conducted in this study highlight the important aspects of how the RRT handoff differs from a handoff elsewhere in the hospital setting. Thus, the handoff process is not a one-size-fits-all approach. Rather, this study suggests that a specific handover tool or communication guide may benefit the best care for these patients, a finding in other studies regarding the handoff process.

This study had several limitations. First, because the focus groups consisted of peers within the same profession and institution, participants may have been more reserved in their responses due to social desirability bias.^37^ Second, the authors did not include background characteristics such as racial and ethnic diversity of the focus group participants in their analysis; however, they do not feel that the results would have been different otherwise. Third, although this is a single-center study, the authors suspect the results and conclusions are generalizable to many Children’s Hospitals or other academic institutions with pediatric centers. Third, due to the COVID-19 pandemic, some focus groups took place in-person, whereas others took place remotely, which could affect the quality of the discussion or participants’ comfort levels. We did not observe this during our transcript comparison. Finally, because this study relied on voluntary participation from the different constituent groups, individuals with stronger positive or negative perspectives about the RRT may have self-selected, thus introducing volunteer bias.^38^

**Figure 1.**
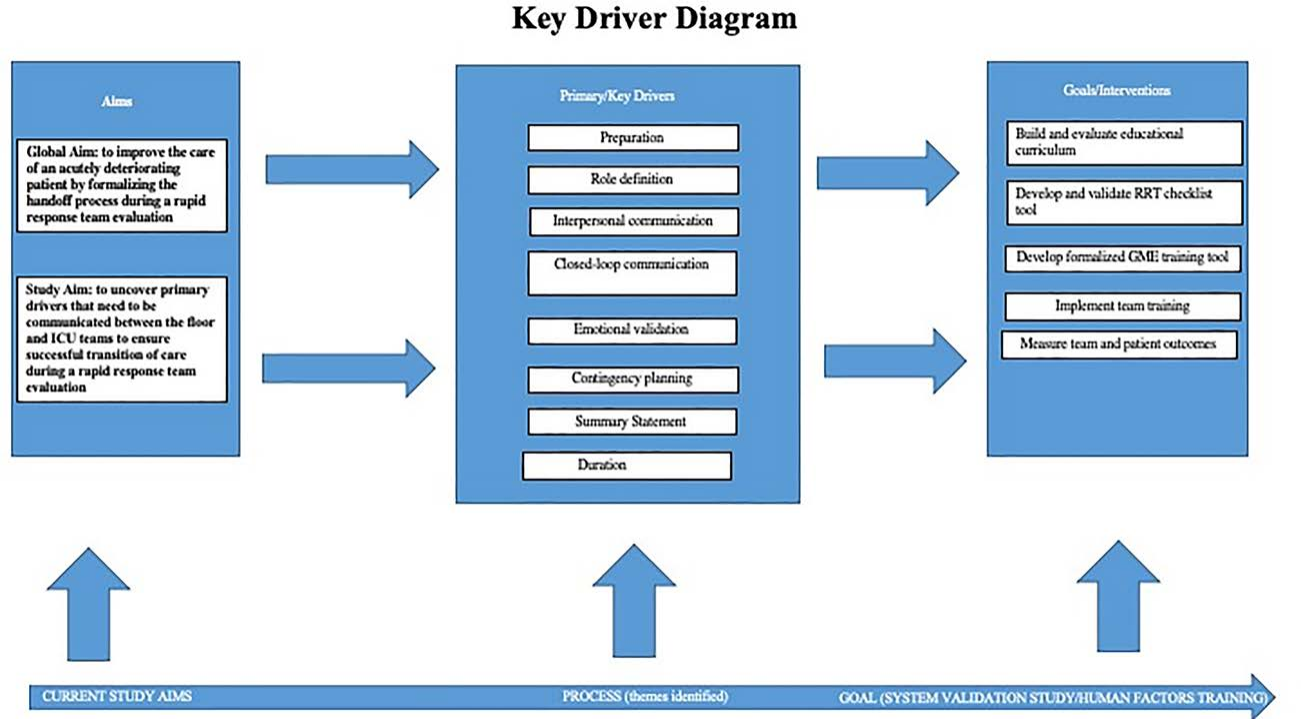
Key Driver Diagram.

The focus of this study was to understand and identify some factors that can compromise patient care during an RRT encounter. Our future research aims include creating and validating a handover tool or aid to improve the RRT encounter and using simulation to teach some of the human factors that affect the transition of care of the clinically deteriorating patient. We have created a Key Driver Diagram highlighting our results and future goals (Fig. 1). The simulation training could be incorporated into the Graduate Medical Education curriculum and used in teamwork training. Once this aid has been created and validated at our institution, we hope to disseminate it to children’s hospitals nationwide, allowing for more efficient evaluation and treatment of the rapidly decompensating patient. This aid could also be used with other clinical assessment tools, such as the Pediatric Early Warning Signs, to help identify children at risk for clinical deterioration and transfer to a PICU. We believe the information reported in this study can ultimately help improve patient care during RRT evaluations.

## ACKNOWLEDGMENTS

The views expressed in this article do not communicate an official position of Children’s Hospital Los Angeles or the University of Southern California.

## DISCLOSURE

The authors have no financial interests to declare in relation to the content of this article.

## Supplementary Material


